# Comparative Genome Analyses of *Vibrio anguillarum* Strains Reveal a Link with Pathogenicity Traits

**DOI:** 10.1128/mSystems.00001-17

**Published:** 2017-02-28

**Authors:** Daniel Castillo, Paul D. Alvise, Ruiqi Xu, Faxing Zhang, Mathias Middelboe, Lone Gram

**Affiliations:** a Marine Biological Section, University of Copenhagen, Helsingør, Denmark; b Department of Biotechnology and Biomedicine, Technical University of Denmark, Kgs Lyngby, Denmark; c Copenhagen Bio Science Park, Denmark; d BGI Park, Yantian District, Shenzhen, China; Dartmouth College

**Keywords:** pan-genome, genomics, virulence factors

## Abstract

*Vibrio anguillarum* is a marine bacterium that can cause vibriosis in many fish and shellfish species, leading to high mortalities and economic losses in aquaculture. Although putative virulence factors have been identified, the mechanism of pathogenesis of *V. anguillarum* is not fully understood. Here, we analyzed whole-genome sequences of a collection of *V. anguillarum* strains and compared them to virulence of the strains as determined in larval challenge assays. Previously identified virulence factors were globally distributed among the strains, with some genetic diversity. However, the pan-genome revealed that six out of nine high-virulence strains possessed a unique accessory genome that was attributed to pathogenic genomic islands, prophage-like elements, virulence factors, and a new set of gene clusters involved in biosynthesis, modification, and transport of polysaccharides. In contrast, *V. anguillarum* strains that were medium to nonvirulent had a high degree of genomic homogeneity. Finally, we found that a phylogeny based on the core genomes clustered the strains with moderate to no virulence, while six out of nine high-virulence strains represented phylogenetically separate clusters. Hence, we suggest a link between genotype and virulence characteristics of *Vibrio anguillarum*, which can be used to unravel the molecular evolution of *V. anguillarum* and can also be important from survey and diagnostic perspectives.

**IMPORTANCE** Comparative genome analysis of strains of a pathogenic bacterial species can be a powerful tool to discover acquisition of mobile genetic elements related to virulence. Here, we compared 28 *V. anguillarum* strains that differed in virulence in fish larval models. By pan-genome analyses, we found that six of nine highly virulent strains had a unique core and accessory genome. In contrast, *V. anguillarum* strains that were medium to nonvirulent had low genomic diversity. Integration of genomic and phenotypic features provides insights into the evolution of *V. anguillarum* and can also be important for survey and diagnostic purposes.

## Abstract (precis)

Comparative genome analysis of strains of a pathogenic bacterial species can be a powerful tool to discover acquisition of mobile genetic elements related to virulence. Here, we compared 28 *V. anguillarum* strains that differed in virulence in fish larval models. By pan-genome analyses, we found that six of nine highly virulent strains had a unique core and accessory genome. In contrast, *V. anguillarum* strains that were medium to nonvirulent had low genomic diversity. Integration of genomic and phenotypic features provides insights into the evolution of *V. anguillarum* and can also be important for survey and diagnostic purposes.

## INTRODUCTION

The genus *Vibrio* belongs to a family of heterotrophic marine bacteria that includes many facultative symbiotic or pathogenic strains (1). Human pathogens include *Vibrio cholerae*, the causative agent of cholera (2), and *V. parahaemolyticus* and *V. vulnificus*, which are responsible for most cases of seafood poisoning (3). However, *Vibrio* infections are also common in marine organisms, as demonstrated by reports of *V. coralliilyticus* being capable of killing coral tissue (4), and several species of *Vibrio* are also of major concern in the aquaculture industry (5, 6). *Vibrio* (*Listonella*)* anguillarum* is the causative agent of a fatal hemorrhagic septicemic disease (vibriosis) and is one of the most important pathogens in the aquaculture and larviculture industry, infecting ~50 species of fish, molluscans, and crustaceans (7). Twenty-three different serotypes (O1 to O23) have been described, with serotypes O1, O2, and to some extent O3 being associated with fish vibriosis (8, 9). The other *V. anguillarum* serotypes are mostly nonpathogenic and represent environmental strains isolated from seawater, plankton, and sediment.

Although the mechanism of pathogenesis of *V. anguillarum* is not completely understood, virulence-related factors have been identified and include chemotaxis and motility (10, 11), adhesion (12), invasion (13, 14), iron sequestration (15, 16), and secretion of extracellular enzymes (17, 18). Several putative virulence genes have been detected in the genome of *V. anguillarum* strain H775-3 (the pJM1-cured strain of 775), including genes encoding exotoxins, adherence/colonization factors, invasion, capsule and cell surface components, and an iron uptake system (19, 20). The first complete genome sequence of *V. anguillarum* strain 775 revealed several genomic features that could explain the pathogenicity of the organism, including the presence of the virulence plasmid pJM1, 10 genomic islands (GIs), potential virulence factors, toxins, and genes evolved in biofilm formation (21). However, genome comparison analyses have demonstrated strain-specific toxins in other virulent *V. anguillarum* strains linked with the absence of the plasmid pJM1, suggesting that *V. anguillarum* strains have evolved different potential virulence mechanisms (21). In contrast, comparative genome analysis of 15 *V. anguillarum* isolates of serotypes O1, O2, and O3 revealed low genetic diversity, and the distribution of putative virulence factors was similar to that in strain 775, suggesting that virulence in *V. anguillarum* is multifactorial (22). The genotypes were compared in a subsequent study to virulence of 15 *V. anguillarum* strains using gnotobiotic European sea bass larvae as the model host (23). No clear correlation between virulence and genotypic was found, and more detailed analyses of whole-genome sequences in comparison with standardized virulence data are required to elucidate the evolution, physiology, and pathogenesis of this bacterium (23). This has been the purpose of the present study.

The microbial genome is divided into core and accessory elements, which combined constitute the pan-genome (24). The core genome includes the pool of genes shared by all the strains of the same bacterial species and typically contains genes required for the essential housekeeping functions of the cell. In contrast, the accessory genome comprises genes found in only some strains and includes genes acquired by horizontal gene transfer events (25). This strain-specific genome could be involved in functions related to pathogenicity, such as niche adaptation (26), antibiotic resistance (27), or production of strain-specific virulence factors (28), which are known to reside within genomic islands (29). Here, we whole-genome sequenced 26 *V. anguillarum* strains isolated from different geographic localities and from different years and hosts. We also included the genomes of two previously sequenced strains, 775 (21) and NB10 (30), and investigated the core genome and the accessory genome to identify virulence genes and explain differences in virulence potential among *V. anguillarum* strains. Our genomic analyses were compared to virulence of the strains as determined in cod, turbot, and halibut larval models (31).

## RESULTS

### Larval mortality caused by *V. anguillarum* strains.

Infection trials with cod, turbot, and halibut larvae divided the 28 strains into four groups of high, medium, low, or no mortality (31) (see Table S1 in the supplemental material).

10.1128/mSystems.00001-17.4 TABLE S1  Average mortality of fish larvae following infection with *Vibrio anguillarum* at high dose (HD) and low dose (LD). Download TABLE S1, DOCX file, 0.1 MB. Copyright © 2017 Castillo et al. 2017 Castillo et al. This content is distributed under the terms of the Creative Commons Attribution 4.0 International license.

### Genome features of *V. anguillarum* strains.

The chromosome sizes of the 28 *V. anguillarum* strains ranged from 3.06 to 3.34 and 0.99 to 1.12 Mb for chromosomes I and II, respectively. The GC contents ranged from 44.0 to 44.8% and 43.6 to 44.1% for chromosomes I and II, respectively. The plasmid pJM1 was found in 17 of the 28 strains (Table 1; see Table S2 in the supplemental material). A total of 3,334 to 3,767 coding sequences (CDS) were predicted per strain (both chromosomes) (Table S2).

10.1128/mSystems.00001-17.5 TABLE S2  Genomic overview of the *V. anguillarum* strains analyzed in this study. Download TABLE S2, DOCX file, 0.1 MB. Copyright © 2017 Castillo et al. 2017 Castillo et al. This content is distributed under the terms of the Creative Commons Attribution 4.0 International license.

**TABLE 1:** Overview of the *V. anguillarum* strains analyzed in this study

Strain	Origin	Yr of isolation	Isolate host	Serotype	Plasmid pJM1	Accession no.	
						Chromosome I/II	Plasmid
4299	Norway	Unknown	Unknown	O2b	−	CP011458/CP011459	
87-9-116	Finland	1987	Atlantic salmon	O1	−	CP010044/CP010045	
87-9-117	Finland	1987	Rainbow trout	O1	+	CP010046/CP010047	CP016253
90-11-286	Denmark	1990	Rainbow trout	O1	−	CP011460/CP011461	
90-11-287	Denmark	1990	Rainbow trout	O1	+	CP011475/CP011476	CP016254
91-7-154	Denmark	1991	Turbot	O1	+	CP010082/CP010083	CP016255
178/90	Italy	Unknown	Sea bass	O1	+	CP011470/CP011471	CP016257
601/90	Italy	Unknown	Sea bass	O1	+	CP010076/CP010077	CP016259
775	United States	Unknown	Coho salmon	O1	+	CP002284/CP002285	AY312585
9014/8	Denmark	1990	Rainbow trout	O1	+	CP010038/CP010039	CP016262
DMS21597	Norway	Unknown	Atlantic cod	O2	−	CP010084/CP010085	
H610	Norway	Unknown	Atlantic cod	O2a	−	CP011462/CP011463	
NB10	Sweeden	Unknown	Unknown	O1	+	LK021130/LK021129	LK021128
PF4	Chile	2004	Salmon salar	O3	−	CP010080/CP010081	
PF7	Chile	2004	Salmon salar	O3	−	CP011464/CP011465	
PF430-3	Chile	2013	Unknown	O3	−	CP011466/CP011467	
S2 2/9	Denmark	Unknown	Rainbow trout	O1	−	CP011472/CP011473	
VA1	Greece	2014	Sea bass	O1	+	CP010078/CP010079	CP016265
6018/1	Denmark	Unknown	Rainbow trout	O1	+	CP010291/CP010292	CP016260
VIB18	Denmark	Unknown	Rainbow trout	O1	+	CP011436/CP011437	CP016266
261/91	Italy	Unknown	Sea bass	O1	+	CP010032/CP010033	CP016258
A023	Spain	Unknown	Turbot	O1	−	CP010036/CP010037	
LMG12010	Unknown	Unknown	Unknown	O1	+	CP011468/CP011469	CP016263
T265	United Kingdom	Unknown	Atlantic salmon	O1/VaNT1	+	CP010040/CP010041	CP016263
51/82/2	Germany	Unknown	Rainbow trout	O1	−	CP010042/CP010043	
VIB93	Denmark	1985	Rainbow trout	O1	+	CP011438/CP011439	CP016267
91-8-178	Norway	1991	Turbot	O1	+	CP010034/CP010035	CP016256
Ba35	United States	Unknown	Sockeye salmon	O1/VaNT1	+	CP010030/CP010031	CP016261

### The *Vibrio anguillarum* pan-genome.

**FIG 1:**
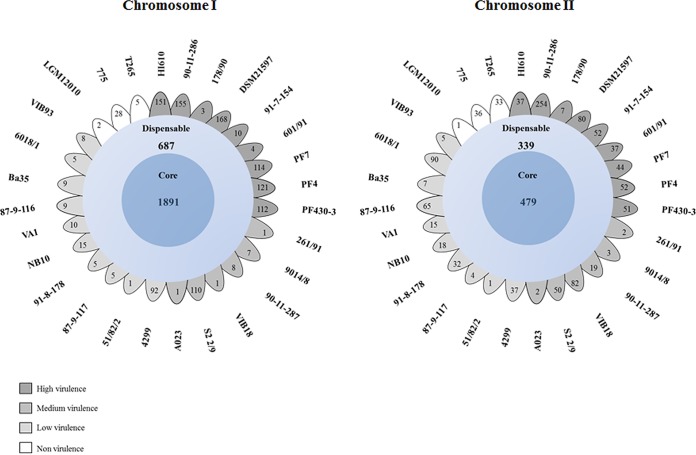
The pan-genome of *V. anguillarum*. The flower plots represent the number of shared (core) and specific (accessory/dispensable) genes based on cluster orthologs for each chromosome. Petals display numbers of strain-specific genes found in each genome of *V. anguillarum* strains with core gene numbers in the center. The gray colors indicate the virulence category as found in three fish larva model systems (31).

To determine an overall approximation of the total gene pool for *V. anguillarum* based on the sequenced genomes, we calculated the pan-genome using the EDGAR software platform. The gene repertoire of the *V. anguillarum* pan-genome increased with each addition of a new genome and had at least 3,973 and 1,932 genes for chromosomes I and II, respectively (see Fig. S1 in the supplemental material). In contrast to this increase, the *V. anguillarum* core genome decreased with the addition of each new genome, as expected (Fig. S1). The* V. anguillarum* average gene contents were 1,891 and 479 genes for chromosomes I and II, respectively (Fig. 1; Fig. S1). These open reading frames (ORFs) belonging to the core genome were assigned to putative functional categories using the Clusters of Orthologous Groups of Proteins (COG) database (Fig. S1B). Approximately 55.4 and 17.3% of the predicted genes in the core genome were dedicated to metabolic functions for chromosomes I and II, respectively. Of these genes, 36.2 and 13.7% were split between cellular process/signaling functions and information storage/processing functions for chromosomes I and II, respectively. Finally, functions of the predicted genes in the remaining 8.4 and 69% of the core genome were assigned as uncharacterized proteins for chromosomes I and II, respectively (Fig. S1B).

10.1128/mSystems.00001-17.1 FIG S1  *V. anguillarum* pan-genome and core and accessory genome evolution according to the number of sequenced genomes. (A) Total numbers of genes (pan-genome), shared genes (core genome), and unique genes (accessory genome) for a given number of genomes sequentially added. (B) COG subcategories of predicted genes within the core genomes of *V. anguillarum* for chromosomes I and II. Each category or subcategory is graphed as a percentage of the total number of genes in the core genome. Download FIG S1, DOCX file, 0.5 MB. Copyright © 2017 Castillo et al. 2017 Castillo et al. This content is distributed under the terms of the Creative Commons Attribution 4.0 International license.

The remaining 1,153 ORFs (chromosome I) and 1,117 ORFs (chromosome II) were defined as the *V. anguillarum* accessory genome (Fig. 1). The number of nonduplicated unique genes in each *V. anguillarum* strain varied from 1 to 168 for chromosome I and from 1 to 254 for chromosome II. The *V. anguillarum* strains 90-11-286, DSM21597, HI610, PF4, PF430-3, PF7, and S2 2/9 had the largest numbers of accessory genes (1,499 for both chromosomes).

### Distribution of virulence factors.

Several virulence-associated genes have been described in *V. anguillarum* strain 775 (21). More than 90% of these virulence genes were present in all strains (see Table S3 in the supplemental material). Genes involved in iron transport, metalloproteases, motility, chemotaxis, type IV pilus, and quorum sensing were found in all strains (Table S3). However, *V. anguillarum* strains PF4, PF430-3, PF7, and S2 2/9 lacked the type VI secretion system present on chromosome I. Moreover, none of the *V. anguillarum* strains had the transcriptional regulator *hylU* or the unknown protein related to catechol siderophore metabolism positioned in chromosome II. Several strains did not have genes for secreted lipase and collagenase (Table S3). All strains in which the plasmid pJM1 was found carried genes related to anguibactin and iron metabolism (Table S3).

10.1128/mSystems.00001-17.6 TABLE S3  Distribution of virulence-related genes in *V. anguillarum* strains. Download TABLE S3, XLSX file, 0.1 MB. Copyright © 2017 Castillo et al. 2017 Castillo et al. This content is distributed under the terms of the Creative Commons Attribution 4.0 International license.

**FIG 2:**
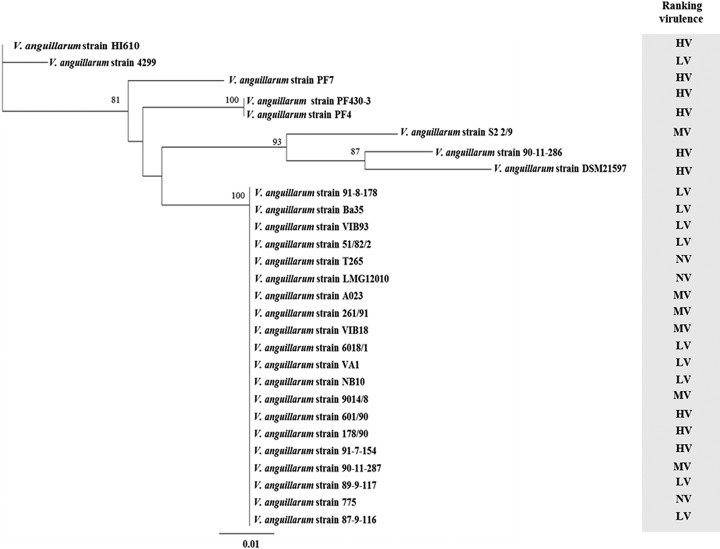
Phylogenetic tree of 163 concatenated virulence factors shared by all *V. anguillarum* strains. The phylogenetic tree was constructed based on the maximum likelihood algorithm, using a concatenated alignment of 163 amino acid sequences inferred from putative virulence factors identified in *V. anguillarum* strain 775 (20) (Table S2). Bootstrap values <80% were removed from the tree. The horizontal bar at the base of the figure represents 0.01 substitutions per amino acid site. The virulence ranking of the strains is based on three fish larva models (31). HV, high virulence; LV, low virulence; MV, medium virulence.

Although our results showed a global distribution of homologous virulence factors, nucleotide sequence dissimilarities could be translated to changes in amino acid level, which affect the dynamic functions or activities of these pathogenicity factors, leading to the development of a more invasive infection (32–34). To reveal the evolution of the virulence, we inferred the genetic diversity of 163 representative virulence factors shared by the *V. anguillarum* strains using the maximum likelihood algorithm (Fig. 2). *V. anguillarum* strains with medium to low virulence tended to cluster in a homogenous group, and the high-virulence strains 90-11-286, PF4, PF430-3, PF7, DSM21597, and HI610 clustered as separate groups (Fig. 2).

### Phylogenetic relationship.

**FIG 3:**
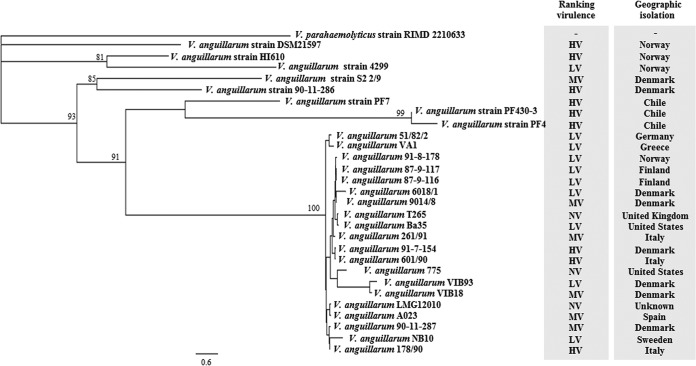
Core genome phylogeny of *V. anguillarum* strains. The maximum likelihood tree was obtained from a concatenated nucleotide sequence alignment of the orthologous core genes (1,723 genes for both chromosomes) for the 28 *V. anguillarum* strains. The virulence properties of the strains and geographical places of isolation were added to improve comparison. Bootstrap values of <80% were removed from the tree. The horizontal bar at the base of the figure represents 0.6 substitution per nucleotide site. The virulence ranking of the strains is based on three fish larva models (31). HV, high virulence; LV, low virulence; MV, medium virulence.

In order to examine potential associations between the core genome composition and the virulence properties, the phylogeny of *V. anguillarum* strains was inferred by constructing a genome-relatedness maximum likelihood tree using orthologous alignment of 2,370 protein-coding genes (both chromosomes) of the core genome (Fig. 1). The evolutionary tree displayed different cluster patterns, which varied in the levels of diversity. Interestingly, 20 of the 28 strains that were medium to nonvirulent in the larval assays grouped with a very low genetic diversity (Fig. 3), with the exception of strains 601/90, 178/90, and 91-7-154, which were highly virulent in the larval systems (30) (Table S1). In contrast, *V. anguillarum* strains 90-11-286, PF4, PF430-3, PF7, DSM21597, and HI610, all of which were highly virulent, each constituted a separate cluster (Fig. 3). *V. anguillarum* strain DSM21597 was the most distant lineage in the phylogenetic tree. However, strains S2 2/9 and 4299, which were medium to low virulence, grouped with strains 90-11-286 and HI610, respectively (Fig. 3). These findings suggest an association between virulence and shared gene content. Moreover, the core phylogenetic tree indicated a geographic association among the most genetically diverse *V. anguillarum* strains. For example, the *V. anguillarum* strains from Chile, PF4, PF430-3, and PF7, shared a common ancestor. Similarly, strains HI610 and 4299, isolated in Norway, and strains S2 2/9 and 90-11-286, isolated in Denmark, clustered according to the geographic locality of isolation (Fig. 3).

### GIs, prophages, and strain-specific genes.

**FIG 4:**
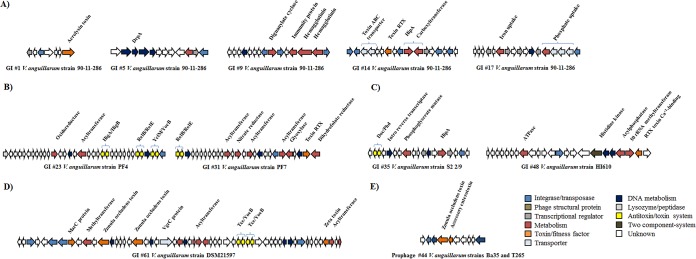
Schematic representation of accessory elements carrying virulence or fitness factors in the *V. anguillarum* strains. (A) Genomic islands in strain 90-11-286. (B) Genomic islands in strains PF4 and PF7. (C) Genomic islands in strains S2 2/9 and HI610. (D) Genomic island in strain DSM21597. (E) Prophage-related elements in *V. anguillarum* strains T265 and Ba35 that contain a gene related to zonula occludens toxin (Zot). The positions of GIs and prophage-like elements are shown in Tables S4 and S7. The colors were assigned according to the possible role of each ORF as shown in the figure.

Ten genomic islands (GIs) have been described in *V. anguillarum* strain 775 (21), and their distribution in our collection was determined (see Fig. S2 in the supplemental material). All GIs (GIs 1 through 10) were found only in strains T265 and 775. Interestingly, the specific GIs 4, 6, 7, 8, and 10 were present in 82, 68, 64, 57, and 55% of the strains in the *V. anguillarum* collection, respectively (Fig. S2). For the purpose of this study, a GI was defined as a specific genomic region containing five or more ORFs (>5 kb). We detected a total of 64 strain-specific GIs between 6 and 132.1 kb for strains 90-11-286, PF4, PF430-3, PF7, HI610, DSM21597, 4299, and S2 2/9, all associated with transposases or integrases (see Table S4 in the supplemental material). Six of these nine strains were all highly virulent in cod, turbot, and halibut larva systems (31) (Table S1). A total of 1,067 strain-specific ORFs were found in these GIs. The G+C contents ranged from 26.2 to 44.9% for GIs in chromosome I and 32.0 to 52.9% for GIs in chromosome II (Table S4). Genes related to toxins, fitness factors, modification-restriction systems, antitoxin-toxin systems, transport, and metabolism were found in the GIs. *V. anguillarum* strain 90-11-286 had 21 specific GIs: one of them had an aerolysin toxin (GI 1), one contained diaguanylate cyclase and hemagglutinin genes (GI 9), and two harbored genes related to toxin RTX and toxin ABC transporter (GI 14) and iron and phosphate uptake systems (GI 17) (Fig. 4A). This strain contained a GI (GI 5) encoding a DprA protein, which has been associated with DNA transport and natural transformation competence (Fig. 4A).

10.1128/mSystems.00001-17.2 FIG S2  Distribution of genomic islands (GIs) previously identified in *V. anguillarum* strain 775. Shown is a graphic representation of the distribution of 10 GIs (GIs 1 to 10) in the *V. anguillarum* collection. Black and white squares represent the presence and absence of GI, respectively. Download FIG S2, DOCX file, 0.3 MB. Copyright © 2017 Castillo et al. 2017 Castillo et al. This content is distributed under the terms of the Creative Commons Attribution 4.0 International license.

10.1128/mSystems.00001-17.7 TABLE S4  Features of the 64 strain-specific genomic islands in the *V. anguillarum* genomes. Download TABLE S4, DOCX file, 0.1 MB. Copyright © 2017 Castillo et al. 2017 Castillo et al. This content is distributed under the terms of the Creative Commons Attribution 4.0 International license.

*V. anguillarum* strain PF4 had a GI of 24.8 kb that encoded three antitoxin-toxin systems and one oxidoreductase gene (GI 23) (Fig. 4B). Also, strain PF7 had a GI that encoded many acyltransferases, RTX toxin, nitrate reductase, and one glyoxalase gene related to antibiotic resistance (GI 31) (Fig. 4B). One GI of strain S2 2/9 carried genes coding for HipA protein and an antitoxin-toxin system (GI 35) (Fig. 4C). Strain HI610 harbored a GI with the presence of 50 rRNA methyltransferase, which is related to antibiotic resistance and RTX toxin Ca^2+^-binding protein (GI 48) (Fig. 4C). The GI of strain DSM21597 had genes encoding zonula occludens toxins (Zots) and the protein MarC related to resistance to antibiotics (Fig. 4D). Many other GIs harbored genes of ecological interest, but specific details for each of these are out of the scope of this article.

**FIG 5:**
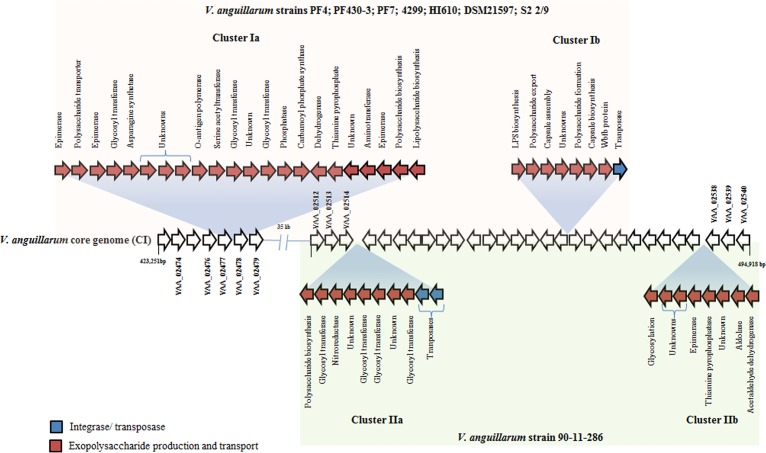
Schematic representation of accessory gene clusters in *V. anguillarum* strains PF4, PF430-3, PF7, 4299, HI610, DSM21597, S2 2/9, and 90-11-286. The position of the core genome was assigned according to *V. anguillarum* strain 775.

Strains that grouped in different phylogenetic lineages possessed a new set of gene clusters participating in the biosynthesis and transport of exopolysaccharides (Fig. 5). Strains PF4, FP430-3, PF7, HI610, 4299, S2 2/9, and DMS21597 had two clusters of 18.6 kb and 9.1 kb (one of them linked to transposases), encoding polysaccharide transports, glycosyltransferases, capsule assembly, and O-antigen polymerase (Fig. 5). Also, *V. anguillarum* strain 90-11-286 contained two gene clusters of 7.5 kb and 8.5 kb at chromosome I that contain genes related to glycosyltransferases, epimerases, and polysaccharide biosynthesis (Fig. 5).

Finally, a new set of specific putative virulence factors were identified in strains 90-11-286 and 91-7-154 (see Table S5 in the supplemental material). *V. anguillarum* strain 90-11-286 had three additional hemagglutinin proteins, one toxin Fic protein, and one cytotoxic necrotizing factor 2 protein (Table S5). Strain 91-7-154 had a Zot. Interestingly, noticeable amino acid similarities (>74%) of these virulence factors were found to those of other *Vibrio* species (*V. cholerae*, *V. ordalii*, and *V. harveyi*), indicating that the genes coding for these proteins may have extrachromosomal origin (Table S5).

10.1128/mSystems.00001-17.8 TABLE S5  Distribution of virulence factors not associated with prophages related sequences or genomic islands in *V. anguillarum* strains. Download TABLE S5, DOCX file, 0.1 MB. Copyright © 2017 Castillo et al. 2017 Castillo et al. This content is distributed under the terms of the Creative Commons Attribution 4.0 International license.

### Prophages.

Fifty-five different prophage-related elements were detected in the *V. anguillarum* genome sequences: of these, 9% were intact prophages, and the rest were defined as a “cryptic” or incomplete prophages. Both types of prophages were found in chromosomes I and II (see Tables S6 and S7 in the supplemental material). Specifically, 40 (72%) unique phage-related sequences between 5.3 and 49.2 kb were specific in 17 of the 28 *V. anguillarum* strains (Table S6). On other hand, 15 phage-related sequences (28%) were shared in 24 out of 28 *V. anguillarum* strains independently of locality and year of isolation (Table S7). Investigation of the presence of virulence or fitness factors encoded inside these sequences showed that *V. anguillarum* strains T265 and Ba35 carried a prophage-like element of 9.2 kb linked to a Zot-like toxin (Fig. 4E; Table S7 [prophage 43]).

10.1128/mSystems.00001-17.9 TABLE S6  Unique prophage-related sequences distributed in *V. anguillarum* strains. Download TABLE S6, DOCX file, 0.1 MB. Copyright © 2017 Castillo et al. 2017 Castillo et al. This content is distributed under the terms of the Creative Commons Attribution 4.0 International license.

10.1128/mSystems.00001-17.10 TABLE S7  Shared prophage-related sequences in *V. anguillarum* strains. Download TABLE S7, DOCX file, 0.1 MB. Copyright © 2017 Castillo et al. 2017 Castillo et al. This content is distributed under the terms of the Creative Commons Attribution 4.0 International license.

## DISCUSSION

The 28 *V. anguillarum* strains analyzed in the present study represent the largest collection of genome-sequenced strains for this fish-pathogenic bacterium. The multiscale comparative approach used in this work provides insights into the diversity of *V. anguillarum* strains (Fig. 1 to 4). Identification and characterization of accessory genome included genes that confer resistance to antibiotics and encode toxins and/or genes that improve the fitness of the organism, which may have been acquired via lateral gene transfer (35) (Fig. 4). In addition, core genome diversity indicated that the most virulent strains grouped in different genetic clusters (Table S1; Fig. 3). Thus, altogether our data indicate that virulence is multifactorial in *V. anguillarum* and that both the core and accessory genomes affect the pathogenicity of this *Vibrio* species. Similarly, the accessory and core genomes are significant sources of virulence-associated genes in *Klebsiella pneumoniae* (36), *Escherichia coli* (37), *Staphylococcus aureus* (38), and *Pseudomona aeruginosa* (39).

The plasmid pJM1 has been described as an important virulence factor in *V. anguillarum* (21, 40). However, *V. anguillarum* strains 90-11-286, PF4, PF7, and HI610, which were highly virulent against fish larvae, did not contain the plasmid pJM1 (Table 1; Table S1), but they had a functional vanchrobactin locus (not interrupted by a transposon) in the bacterial chromosome (31). This observation is in accordance with a previous study in which virulent pJM1-deficient strains did not carry the anguibactin system but produced the chromosomally encoded siderophore vanchrobactin, which is potentially a virulence factor (23, 41, 42). Thus, our results suggest that the presence or absence of the pJM1 plasmid is not an essential factor for *V. anguillarum* to cause disease in fish larvae.

The pan-genome analysis revealed that *V. anguillarum* contained a core genome of 2,370 nonduplicated ORFs for both chromosomes (Fig. 1; Fig. S1). This level of core gene content is higher than that reported for *V. mimicus* (43) but lower than those in *V. parahaemolyticus* (44) and *V. cholerae* (45). When the *V. anguillarum* core genome was used to determine phylogeny, the 28 *V. anguillarum* strains clustered in 5 groups, and six of the nine most virulent strains were found in separate clusters (Fig. 3). In contrast, most of the strains that showed moderate to no virulence in our larval systems shared a very similar backbone, and hence probably all originated from a common ancestor (Fig. 3). These characteristics could be relevant for understanding the relationship between the core genome diversity and the influence of acquired mobile elements on pathogenicity in *V. anguillarum*. We speculate that these phylogenetically distant bacteria could occupy different niches in fish farms and that the acquisition of GIs may vary in these aquatic systems and be influenced by the genetic background. Interestingly, three *V. anguillarum* strains (91-7-154, 601/90, and 178/90) that displayed high-virulence properties in the larval systems clustered with the strains that showed medium- or low-virulence properties (Fig. 3; Table S1). This observation allows us to suggest that specific mutations in the core genome may be linked to their high-virulence phenotypes.

Both chromosomes contained strain-specific elements and new virulence genes as a result of insertion of different genomic islands, prophages, and/or acquisition of other mobile genetic elements (described below) (Tables S4, S6, and S7). These results are in contrast to previous analysis in *V. antiquaries* (46), or *V. mimicus* (43), where chromosome II represents a collection of accessory elements and likely participate in the adaptation to different niches, having a critical role in the speciation and evolution of the genus *Vibrio* (47). However, our results indicated that chromosomes I and II both have genome plasticity (Fig. 1; Fig. S1), leading us to suggest that both chromosomes are involved in and driving the evolution in *V. anguillarum*.

To capture the dynamic nature of virulence gene repertoires across *V. anguillarum*, we screened for >200 virulence related genes (21) (Table S3). A total of 163 genes were present in all of the strains (belonging to core genome), and phylogenetic relationships displayed a functional divergence in six out of nine of the most virulent strains (Fig. 2). This diversification leads us to suggest that virulence activity is under strong selection, affecting the dynamic functions or activities of these proteins, leading to the development of a stronger interaction with the host in the different steps of infection, as has been proposed for *Pseudomonas syringae* (32) and *P. aeruginosa* (48).

Genomic islands contribute to the evolution and diversification of microbial communities (49). We found 64 GIs that belonged to the accessory genome among six out of the nine most virulent *V. anguillarum* strains (Table S4). *V. anguillarum* strain 90-11-286 was highly virulent in the larval models (Table S1) and harbored GIs carrying a diversity of virulence factors (Fig. 4A). For example, one GI encoded the channel-forming toxin aerolysin, which has been associated with diarrheal diseases and deep wound infections, by interacting with eukaryotic cells and aggregating to form pores, leading to the destruction of the membrane permeability barrier and osmotic lysis (50). A second GI had a diguanylate cyclase gene, which affects the adhesive and invasive capabilities of the human pathogen *Porphyromonas gingivalis* (51). Finally, a third GI contained a hemolysin toxin, RTX, associated with toxin ABC transporter (52). Interestingly, this strain harbored the highest number of accessory genes in both chromosomes (Fig. 1; Fig. S1), and this feature could be associated with the presence of the protein DprA (Fig. 4A), which participates in uptake, transport, and protection of DNA in the natural transformation process (53).

Strains PF4, PF7, and S2 2/9 harbored GIs that encoded several acyltransferases and toxin-antitoxin systems (Fig. 4B and C). Acyltransferases are enzymes that transfers acyl groups to specific targets and may be an important factor regulating quorum-sensing virulence-related phenotypes, including the production of virulence factors, motility, and biofilm formation (54). Toxin-antitoxin systems, which were originally linked to the plasmid maintenance and stabilization of the bacterial chromosome, are now known to be involved in general stress response (55), persistence (56), biofilm formation (57), and virulence capacity of pathogenic bacteria (58). Finally, strains HI610 and DSM21597 exhibited GIs which encode toxins and resistance to reactive oxygen species (Fig. 4C and D). Genomic island in strain HI610 had a 50 rRNA methyltransferase, which contribute to the virulence in *Staphylococcus aureus* by conferring resistance to oxidative stress (59) and hemolysin toxin RTX Ca^2+^ binding (52). Strain DSM21597 had two zonula occludens toxins (Zot), described previously in *V. cholerae*, whose function is to increase intestinal permeability by interacting with a mammalian cell receptor, with subsequent activation of intracellular signaling leading to the disassembly of the intercellular tight junctions (60, 61).

A set of prophage-related elements were identified in all 28 *V. anguillarum* strains (Table S6 and S7). Two prophage-like elements in the strains T265 and Ba35 contained Zot genes, which shared homology with *V. cholerae* (Fig. 4E). The presence of this toxin has also been documented in a prophage genome in *V. coralliilyticus* (62). However, recent studies have indicated that prophage elements may provide a benefit on virulence or fitness evolution, even if they do not carry virulence factors (63). Thus, this finding could be a starting point for future experimental studies on the role of bacteriophages as a potential central driver of pathogenicity in *V. anguillarum*.

Comparative genome analysis also revealed that most virulent strains 90-11-286, PF4, PF430, PF7, HI610, and DSM21597, and the low-virulence strain 4299 carried a new set of gene clusters related to biosynthesis, modification, and transport of exopolysaccharides (Fig. 5). In contrast to the limited diversity observed in this region among the 21 remaining strains, it clearly indicated that these clusters have been horizontally transferred. The existence of these accessory genes is regarded as essential virulence factor in *Burkholderia pseudomallei* (64) and *V. cholerae* (45). More importantly, the presence of these genes has been associated with the modification of capsule polysaccharide content and evasion of immune response (65).

Unlike *Salmonella enterica* serovar Typhi (66), *Bacillus anthracis* (67) and *V. parahaemolyticus* O3:K6 (68), which showed low genetic diversity, *V. anguillarum* offered an example of how lateral gene transfer has an important role in accessorizing the genome, providing genes essential for pathogenicity or fitness (Fig. 4; Table S5). Taken altogether, we propose a hypothetical model of evolution in *V. anguillarum* occurring in distinct phylogenetic groups, which shows that the high-virulence properties of some strains were obtained mainly via acquisition of pathogenic genomic islands occurring in the natural environment (see Fig. S3 in the supplemental material). It should be noted that the stability and transmission of these GIs were speculative. For example, GIs 4 and 6 detected previously in strain 775 were present in 82 and 68% of the strains, respectively, independently of the geographic localities of isolation (Fig. S2). Thus, we assumed a vertical transmission and loss of these specific GIs in some phylogenetic clusters. In contrast, GIs 3 and 5 were presented in 7% and 14%, respectively (Fig. S2), and their transmission could be horizontal for those strains. Similarly, we hypothesized that temperate bacteriophages infected putative *V. anguillarum* genetic ancestors (e.g., Pp41) (Table S7) and consequently the prophage-related elements were transmitted vertically, while other bacteriophages could be strain specific (e.g., Pp 16). Altogether, this study proposes that GIs and prophage-related elements outside the core genome may be a driving force in diversity and pathogenicity of *V. anguillarum* (Fig. S3).

10.1128/mSystems.00001-17.3 FIG S3  Hypothetical evolution pathway in *V. anguillarum*. The model of *V. anguillarum* evolution suggests insertions and deletion of genomic islands (Table S4) and infection by bacteriophages (Tables S6 and S7). The graphic representation of the genetic diversity is based on the core genome phylogeny. Putative ancestral strains are indicated as open circles. The virulence ranking of the strains is based on three fish larval models (1). Download FIG S3, DOCX file, 0.4 MB. Copyright © 2017 Castillo et al. 2017 Castillo et al. This content is distributed under the terms of the Creative Commons Attribution 4.0 International license.

*V. anguillarum* is an important part of the autochthonous marine microbial communities with a specific ecological niche, such as fish, where selective pressure may allow acquisition of genetic traits that could increase fitness and virulence potential (69). Data presented here clearly support this view, where genomic islands carrying a suite of virulence genes and other mobile elements are probably driving the pathogenic and/or fitness evolution of *V. anguillarum* (Fig. 4; Tables S4 and S5). It has been suggested that virulence factors have a dual function and are used by pathogens both during the host infection and in environmental adaptation (70). For example, the toxin hemagglutinin in *V. cholerae* has a role in intestinal colonization, but has also recently been implicated in biofilm formation on chitin-containing surfaces in aquatic environment (71). In the same way, *V. antiquarius*, isolated in a deep sea hydrothermal vent, exhibited Zot and RTX toxins (46), indicating a multifaceted role outside the host. Thus, the presence of these genes in the *V. anguillarum* strains (Fig. 4; Tables S4 and S5) suggests a dual role in non-host environments; however, clearly a new outlook is needed for inferring the putative secondary role of pathogenic genes in this bacteria.

### Conclusions.

Using a comparative pan-genomic analysis of *V. anguillarum*, we identified new pathogenic genomic islands, prophages, and virulence factors, suggesting that independent acquisition of these mobile genetic elements could play an important role in the evolution and virulence of *V. anguillarum*. The phylogenetic relationship based on core genome and shared virulence factors revealed different cluster groups, which suggested a possible link with the virulence properties and supported the idea that pathogenicity is also driven by core genome content within this bacterial species. Altogether, the genome sequences analyzed could serve as a reference point for studies of pathogenicity in aquaculture when *V. anguillarum* is present.

## MATERIALS AND METHODS

### Strain selection, medium composition, and growth conditions.

Twenty-eight *V. anguillarum* strains isolated from different geographic localities (>13,000 km), temporal scales (>25 years), and hosts (Table 1) were included in the analyses. The strains were stored at −80°C in LB broth (12106; Mo-Bio) with 15% glycerol. Strains were grown in LB broth and incubated at 22°C with agitation for 24 h (72).

### DNA extraction.

Bacterial DNA from *V. anguillarum* strains was extracted from cells harvested by centrifugation (5000 × *g*, 10 min) using the NucleoSpin tissue kit (Macherey-Nagel). The amount of genomic DNA was measured using a Nanodrop2000 UV-visible light (UV-Vis) spectrophotometer (Thermo Scientific).

### Genome sequencing, assembly, and annotation.

The genomes of 25 *V. anguillarum* strains were sequenced using Illumina HiSeq platform (BGI, China) with paired-end read sizes of 100 bp. Library construction, sequencing, and data pipelining were performed in accordance with the manufacturer’s protocols. The Illumina data were assembled into contiguous sequences using Geneious software (version 9.1.4) (73), and short- and low-coverage contigs were filtered out. The remaining contigs were aligned using chromosomes I and II and plasmid pJM1 of *V. anguillarum* strain 775 as references (GenBank accession no. CP002284.1 [chromosome I], CP002285.1 [chromosome II], and AY312585 [plasmid pJM1]; December 2014). The genome assembly process was performed using the Geneious software version 9.1.4 and assembled into two scaffolds of 35 to 71 contigs with an average coverage of >88× for each isolate. The genome of *V. anguillarum* strain 90-11-286 was already fully sequenced and previously described (74). Annotation of the genomes was done by the NCBI Prokaryotic Genome Automatic Annotation Pipeline (PGAAP) (75). Alternatively, genomic annotation was done by RAST (76) and BaSys (77).

### Identification of genomic islands, prophage-like elements, and virulence factors.

IslandViewer and MAUVE v2.3.1 were used to predict the putative genomic islands (GIs) (78, 79). IslandViewer integrated sequence composition-based genomic island prediction programs, including IslandPath-DIMOB, SIGI-HMM, and the comparative genome-based program IslandPick. The MAUVE alignment procedure allows the detection of unique regions using a comparative genomics approach. Putative virulence genes were predicted using the virulence database MvirDB (80). All predicted genes of the 28 *V. anguillarum* strains were searched against the MvirDB by BLASTP with loose criteria (E value, ≥1E−5; identity, ≥35%; coverage, ≥80%). Also, VirulenceFinder 1.2 (81) was used to screen for putative virulence factors using selected databases from *Escherichia coli*, *Enterococcus*, and *Streptococcus aureus*. Prophage-related sequences were identified and selected by running bacterial genomes in Phage_Finder v2.1 (82) and PHAST (83).

Virulence-related genes and genomic islands (GIs) of *V. anguillarum* strain 775 were used to identify homologs in the* V. anguillarum* genomes by BLAST analyses using the tBLASTn 2/2/25+ tool and an E value threshold of ≤10^−10^ (84). These DNA sequences were verified as reciprocal best hits.

### Pan-genome analysis.

To predict the possible genomic dynamic changes at *V. anguillarum*, EDGAR (85) was used to predict the pan-genome: i.e., to determine the accessory genome (specific genes found in only one genome) and core genome (common genes mutually conserved). Comparative analyses at the protein level were done by an all-against-all comparison of the annotated genomes. The algorithm used was BLASTP with a standard scoring matrix, BLOMSUM62, and an E value cutoff of 10^−4^. All BLAST hits were normalized according to the best score (84). The score ratio value (SRV), which shows the quality of the hit, was calculated by dividing the scores of further hits by the best hit (86). Two genes were considered orthologous when revealing a bidirectional best BLAST hit with single SRV exceeding the predetermined cutoff of 76 (85).

Functional annotation of genes and transposase identification was accomplished by BLASTp alignment of annotated ORFs against the COG database (87) using BLAST+ v2.2.24 (88).

### Phylogenomic tree reconstruction.

To reveal the phylogenetic relationship among *V. anguillarum* strains based on virulence factors, we selected 163 putative pathogenicity genes from *V. anguillarum* strain 775 (21). For each gene, protein sequences were aligned using ClustalW version 2.0 (89), and individual proteins were concatenated to infer phylogeny using maximum likelihood in Geneious version 9.1.4 (73). Similarly, to determine the core genome phylogenetic relationship among *V. anguillarum* strains based on genomic data, we selected a set of orthologous genes shared by all 28 strains and *V. parahaemolyticus* strain RIMD 2210633 (outgroup to root the tree) (1,723 genes present in a single copy, with paralogs not included) using OrthoMCL with an E value cutoff of 10^−10^ (90). The sets of 1,723 single core genes were first aligned at the amino acid level using ClustalW version 2.0 (89) and then back-translated to DNA sequences using PAL2NAL (91). The alignment of all orthologous sequences was concatenated using FASconCAT (92). The gene tree was constructed using PhyML (93).

### Accession number(s).

Accession numbers for chromosomes and plasmids are listed in Table 1.
