# Supplementary material for: Comparative Genome Analyses of Vibrio anguillarum Strains Reveal a Link with Pathogenicity Traits
Source: mSystems. 2017 Feb 28;2(1):e00001-17. doi: 10.1128/mSystems.00001-17 (PMC5347184; doi:10.1128/mSystems.00001-17)
Supplement: TABLE S1 [file sys001172089st4.docx]

**Table 1S**. Average mortality of fish larvae following infection with *Vibrio anguillarum* in high dose (HD) and low dose (LD)

| **Strain** | **High dose** | **Low dose** | **Virulence^a^** |
| --- | --- | --- | --- |
| HI610 | 93 | 69 | H |
| 90-11-286 | 87 | 82 | H |
| 178/90 | 84 | 78 | H |
| DSM21597 | 81 | 67 | H |
| 91-7-154 | 78 | 60 | H |
| 601/91 | 77 | 57 | H |
| PF7 | 78 | 50 | H |
| PF4 | 73 | 51 | H |
| PF430-3 | 78 | 69 | H |
| 261/91 | 77 | 41 | M |
| 9014/8 | 60 | 53 | M |
| 90-11-287 | 63 | 42 | M |
| VIB18 | 62 | 45 | M |
| S2 2/9 | 69 | 32 | M |
| A023 | 47 | 39 | M |
| 4299 | 43 | 34 | L |
| 51/82/2 | 40 | 34 | L |
| 87-9-117 | 64 | 50 | L |
| 91-8-178 | 59 | 41 | L |
| NB10 | 50 | 50 | L |
| VA1 | 54 | 38 | L |
| 87-9-116 | 55 | 38 | L |
| Ba35 | 41 | 22 | L |
| 6018/1 | 36 | 33 | L |
| VIB93 | 48 | 25 | L |
| LMG12010 | 45 | 33 | N |
| 775 | 36 | 22 | N |
| T265  Negative control | 33  18 | 21  18 | N |

^a^Ranking in high (H), medium(M), low (L) or no (N) virulence
