# Supplementary material for: Comparative Genome Analyses of Vibrio anguillarum Strains Reveal a Link with Pathogenicity Traits
Source: mSystems. 2017 Feb 28;2(1):e00001-17. doi: 10.1128/mSystems.00001-17 (PMC5347184; doi:10.1128/mSystems.00001-17)
Supplement: TABLE S2 [file sys001172089st5.docx]

**Table 2S.** Genomic overview of the *V. anguillarum* strains analyzed in this study

| Strain | Genome assembly  Status | Size CI/CII (Mb) | Total genes CI/CII | Total CDS CI/CII | Total pseudogenes CI/CII | %GC  CI/CII | rRNA | tRNA |
| --- | --- | --- | --- | --- | --- | --- | --- | --- |
| 4299 | Draft copy | 3.06/1.10 | 3412 | 3334 | 120 | 44.7/44.3 | 4 | 70 |
| 87-9-116 | Draft copy | 3.06/1.10 | 3635 | 3566 | 67 | 44.6/43.8 | 4 | 62 |
| 87-9-117 | Draft copy | 3.06/1.04 | 3569 | 3510 | 64 | 44.6/43.9 | 4 | 51 |
| 90-11-286 | Complete | 3.05/1.29 | 3883 | 3689 | 55 | 44.7/43.8 | 31 | 107 |
| 90-11-287 | Draft copy | 3.06/1.09 | 3703 | 3645 | 61 | 44.6/44.1 | 4 | 50 |
| 91-7-154 | Draft copy | 3.06/1.10 | 3682 | 3624 | 69 | 44.6/44.2 | 4 | 52 |
| 178/90 | Draft copy | 3.06/1.06 | 3492 | 3435 | 57 | 44.6/44.0 | 3 | 50 |
| 601/90 | Draft copy | 3.06/1.20 | 3832 | 3767 | 78 | 44.6/44.2 | 4 | 57 |
| 775 | Complete | 3.06/0.99 | 3666 | 3406 | 127 | 44.6/44.1 | 20 | 86 |
| 9014/8 | Draft copy | 3.06/1.12 | 3610 | 3534 | 61 | 44.6/43.8 | 5 | 68 |
| DMS21597 | Draft copy | 3.32/1.02 | 3658 | 3593 | 82 | 44.5/44.2 | 4 | 60 |
| HI610 | Draft copy | 3.34/1.02 | 3623 | 3565 | 130 | 44.5/44.4 | 4 | 58 |
| NB10 | Complete | 3.12/1.19 | 3936 | 3750 | 66 | 44.7/43.6 | 25 | 93 |
| PF4 | Draft copy | 3.16/1.02 | 3476 | 3396 | 23 | 44.8/44.0 | 4 | 72 |
| PF7 | Draft copy | 3.12/1.06 | 3501 | 3434 | 41 | 44.8/43.8 | 6 | 57 |
| PF430-3 | Draft copy | 3.19/1.01 | 3443 | 3365 | 15 | 44.8/44.0 | 4 | 70 |
| S2 2/9 | Draft copy | 2.97/1.02 | 3479 | 3434 | 35 | 44.0/43.8 | 4 | 37 |
| VA1 | Draft copy | 3.06/1.06 | 3643 | 3586 | 79 | 44.6/44.0 | 4 | 49 |
| 6018/1 | Draft copy | 3.06/1.12 | 3649 | 3585 | 82 | 44.6/44.1 | 4 | 56 |
| VIB18 | Draft copy | 3.06/1.12 | 3706 | 3626 | 84 | 44.6/43.8 | 4 | 72 |
| 261/91 | Draft copy | 3.06/1.03 | 3595 | 3468 | 67 | 44.6/44.1 | 4 | 52 |
| A023 | Draft copy | 3.06/1.04 | 3489 | 3431 | 72 | 44.7/44.0 | 4 | 50 |
| LMG12010 | Draft copy | 3.06/1.04 | 3572 | 3513 | 65 | 44.6/44.0 | 4 | 51 |
| T265 | Draft copy | 3.06/1.00 | 3563 | 3510 | 70 | 44.6/44.1 | 4 | 47 |
| 51/82/2 | Draft copy | 3.06/1.11 | 3598 | 3536 | 75 | 44.6/44.0 | 3 | 55 |
| VIB93 | Draft copy | 3.06/1.01 | 3611 | 3535 | 65 | 44.6/43.9 | 4 | 68 |
| 91-8-178 | Draft copy | 3.06/1.07 | 3619 | 3554 | 65 | 44.6/43.9 | 4 | 57 |
| Ba35 | Draft copy | 3.06/0.99 | 3546 | 3486 | 64 | 44.6/44.1 | 4 | 52 |
