# Supplementary material for: Comparative Genome Analyses of Vibrio anguillarum Strains Reveal a Link with Pathogenicity Traits
Source: mSystems. 2017 Feb 28;2(1):e00001-17. doi: 10.1128/mSystems.00001-17 (PMC5347184; doi:10.1128/mSystems.00001-17)
Supplement: FIG S1 [file sys001172089sf1.docx]

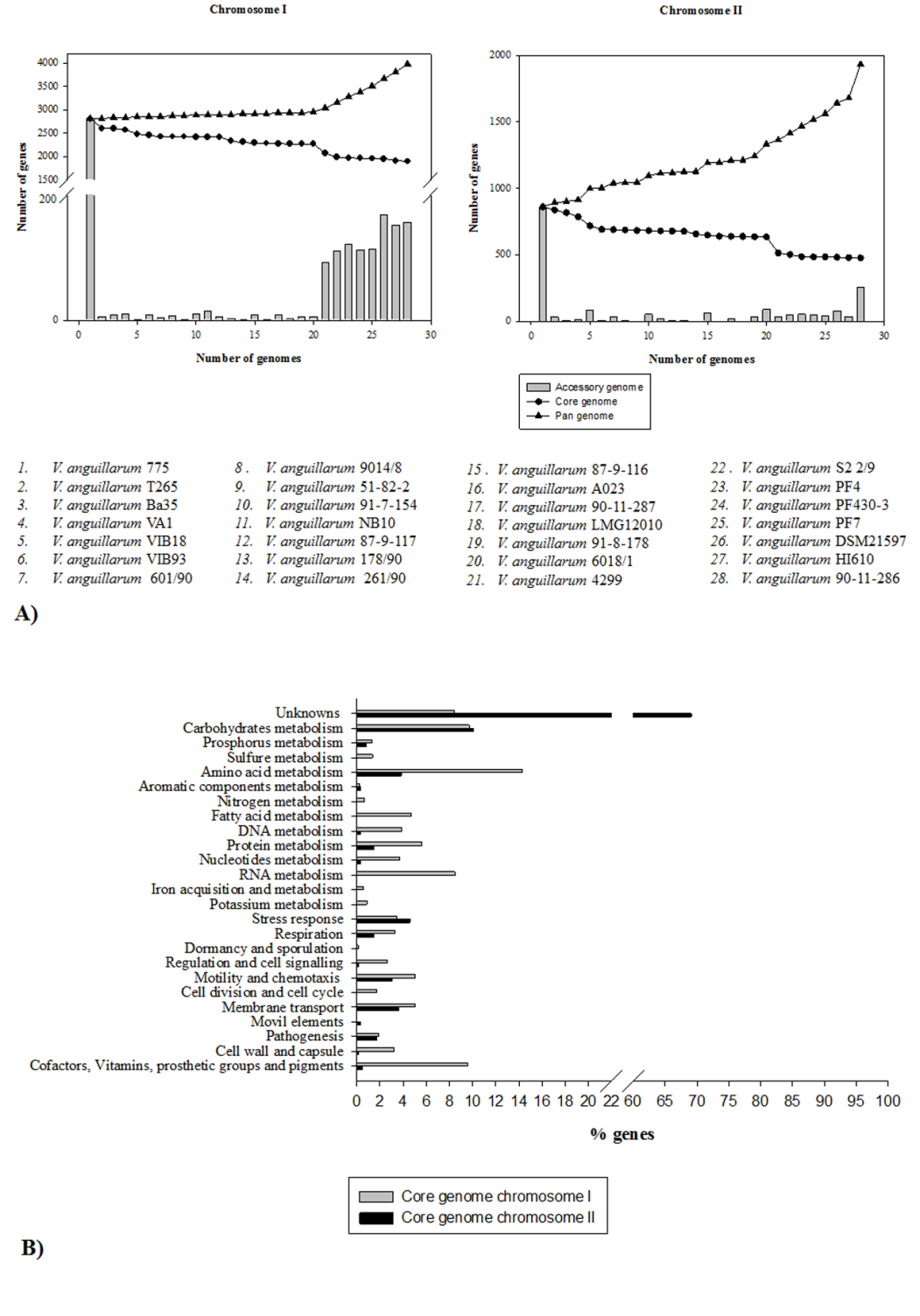


**Fig. 1S.** ***V. anguillarum* pan, core and accessory genome evolution according to the number of sequenced genomes**. A) Total number of genes (pan-genome), shared genes (core genome) and unique genes (accessory genome) for a given number of genomes sequentially added. B) COG subcategories of predicted genes within the core genomes of *V. anguillarum* for chromosomes I and II. Each category or subcategory is graphed as a percentage of the total number of genes in the core genome.
