# Supplementary material for: Comparative Genome Analyses of Vibrio anguillarum Strains Reveal a Link with Pathogenicity Traits
Source: mSystems. 2017 Feb 28;2(1):e00001-17. doi: 10.1128/mSystems.00001-17 (PMC5347184; doi:10.1128/mSystems.00001-17)
Supplement: FIG S2 [file sys001172089sf2.docx]

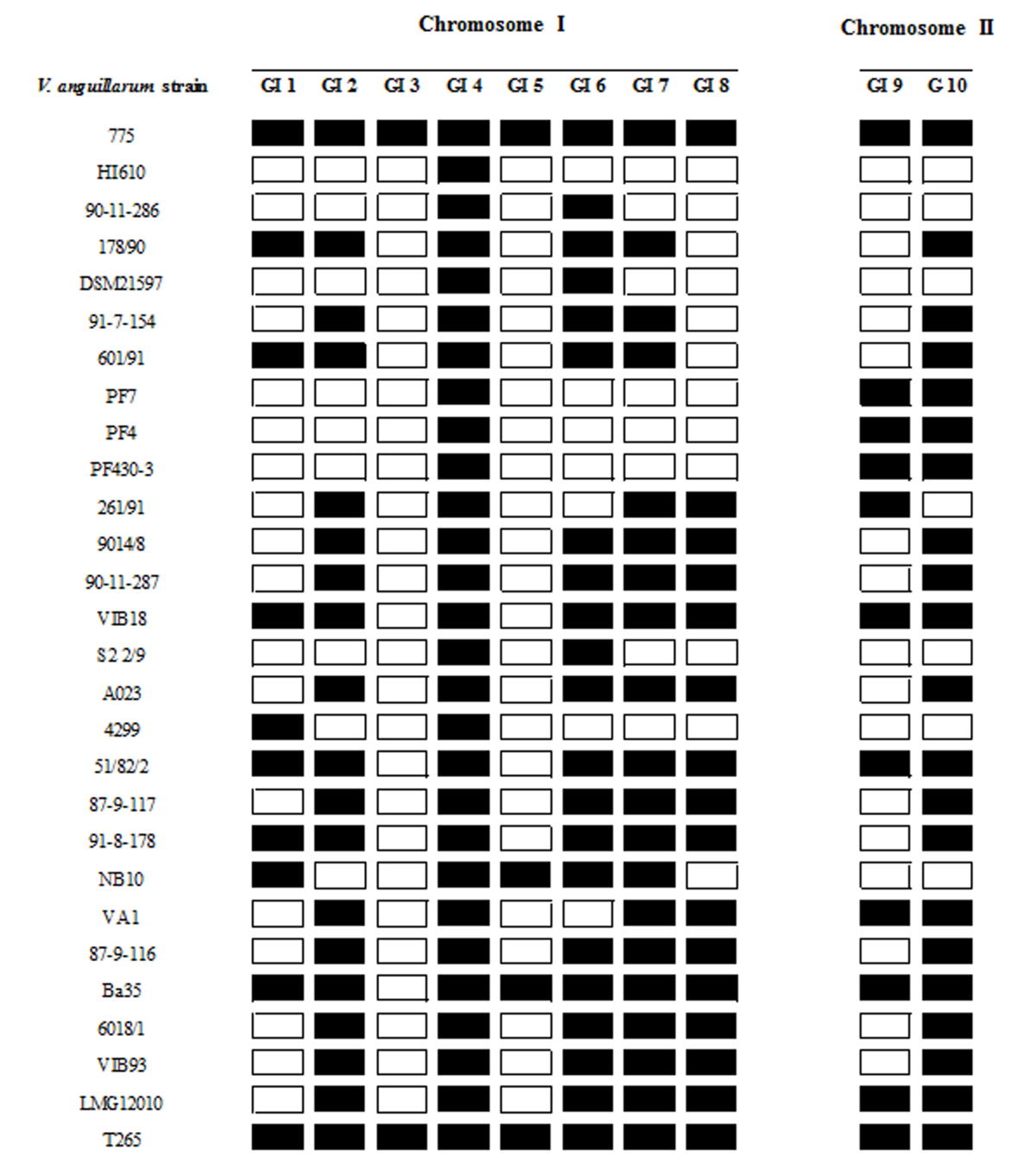


**Fig. 2S**. **Distribution of genomic islands (GIs) previously identified in *V. anguillarum* strain 775**. Graphic representation of the distribution of 10 GIs (GIs 1-10) in the *V. anguillarum* collection. Black and white squares represent presence and absence of GI respectively.
