# Supplementary material for: Comparative Genome Analyses of Vibrio anguillarum Strains Reveal a Link with Pathogenicity Traits
Source: mSystems. 2017 Feb 28;2(1):e00001-17. doi: 10.1128/mSystems.00001-17 (PMC5347184; doi:10.1128/mSystems.00001-17)
Supplement: TABLE S4 [file sys001172089st7.docx]

**Table 4S**. Features of the sixty four strain-specific genomic islands in the *V. anguillarum* genomes

| **Strain** | **GI number** | **Start-end (chromosome)** | **Length (bp)** | **ORFs** | **GC%** | **Main feature genes** |
| --- | --- | --- | --- | --- | --- | --- |
| 90-11-286 | 1 | 35,572-42,604 (CI) | 7,032 | 8 | 38.0 | Aerolysin-toxin; metil-chemotaxis |
|  | 2 | 103,405-111,904 (CI) | 8,500 | 6 | 42.9 | Porin; transporters; polyketide cyclase |
|  | 3 | 270,731-282,248 (CI) | 12,123 | 10 | 31.8 | Retron-type RNA-directed DNA polymerase; unknowns |
|  | 4 | 294,471-300,369 (CI) | 6,169 | 6 | 40.2 | Unknowns |
|  | 5 | 775,074-793,309 (CI) | 18,236 | 12 | 40.0 | DrpA protein; restriction-modification systems; unknowns |
|  | 6 | 1,654,779-1,663,525 (CI) | 8,747 | 6 | 39.8 | Unknowns |
|  | 7 | 1,676,472-1,685,933 (CI) | 9,522 | 9 | 44.9 | Unknowns |
|  | 8 | 2,191,251-2,204,573 (CI) | 13,348 | 11 | 41.5 | Ornithine cyclodeaminase; trypsin; chemotaxis protein; unknowns |
|  | 9 | 2,655,973-2,672,734 (CI) | 16,761 | 16 | 39.2 | Filamentous hemagglutinins; Immunity protein; diguanylate cyclase; unknowns |
|  | 10 | 2,940,019-2,951,876 (CI) | 11,858 | 18 | 37.3 | Oxidoreductase; unknowns |
|  | 11 | 2,960,988-2,985,230 (CI) | 24,243 | 28 | 39.0 | Oxidoreductase; acetyltransferase; unknowns |
|  | 12 | 3,000,038-3,012,959 (CI) | 12,870 | 18 | 41.5 | Glyoxalase; YafQ toxin; unknowns |
|  | 13 | 130,297-136,330 (CII) | 6,034 | 7 | 40.3 | Sphingomyelinase C, unknown proteins |
|  | 14 | 177,941-200,188 (CII) | 22,320 | 16 | 40.0 | Toxin secretion ABC transporter; HLyD secretion protein: HipA protein |
|  | 15 | 206,933-215,043 (CII) | 8,111 | 8 | 38.5 | Transporter; lysine descarboxylase; unknowns |
|  | 16 | 248,905-255,156 (CII) | 6,252 | 8 | 32.0 | Unknowns |
|  | 17 | 420,749-438,474 (CII) | 17,713 | 15 | 52.9 | Ferric reductase; phosphate uptake |
|  | 18 | 436,523-479,541 (CII) | 43,081 | 34 | 42.3 | CRISPR-associated protein aminoacid adenylation; transcriptional regulator; unknowns |
|  | 19 | 1,086,185-1,096,376 (CII) | 10,192 | 9 | 40.9 | Phage-related proteins |
|  | 20 | 1,160,571-1,292,685 (CII) | 132,114 | 136 | 40.3 | Acetyltransferases; lipase; peroxidase; unknowns |
| PF4 | 21 | 2,286,215-2,292,879 (CI) | 6,655 | 6 | 35.8 | Exopolysaccharide biosynthesis |
|  | 22 | 3,006,523-3,012,738 (CI) | 6,216 | 8 | 40.2 | Acyltransferase; unknowns |
|  | 23 | 3,015,959-3,040,779 (CI) | 24,820 | 36 | 26.2 | Antitoxin-toxin systems; unknowns |
|  | 24 | 3,069,748-3,106,184 (CI) | 36,437 | 42 | 38.9 | Acyltransferases; serine/threonine kinases;unknowns |
|  | 25 | 3,139,035-3,157,672 (CI) | 18,612 | 16 | 40.6 | Antitoxin-toxin system; unknowns |
| PF7 | 26 | 1,111,318-1,124,243 (CI) | 12,926 | 12 | 38.5 | Metallo-beta-lactamase; unknowns |
|  | 27 | 2,275,281-2,281,946 (CI) | 6,665 | 6 | 35.7 | Lipopolysaccharide biosynthesis |
|  | 28 | 2,717,102-2,721,580 (CI) | 4,479 | 5 | 39.3 | HipA protein; unknowns |
|  | 29 | 2,768,347-2,825,645 (CI) | 57,298 | 56 | 40.9 | CRISPR associated proteins; Retron-type RNA-directed DNA polymerase; glyoxalases; unknowns |
|  | 30 | 3,020,468-3,027,367 (CI) | 6,900 | 8 | 40.7 | CRISPR associated proteins |
|  | 31 | 3,090,910-3,109,612 (CI) | 18,703 | 27 | 40.2 | Antitoxin-toxin system;acyltransferases RTX toxin; unknowns |
| S2 2/9 | 32 | 428,314-435,850 (CI) | 7,537 | 7 | 33.7 | Export o-antigen; glycosyltransferases |
|  | 33 | 1,013,766-1,018,107 (CI) | 4,342 | 5 | 36.9 | RNA-directed DNA polymerase; unknowns |
|  | 34 | 2,384,763-2,394,176 (CI) | 9,414 | 8 | 36.6 | Thymidylate synthase; unknowns |
|  | 35 | 2,803,380-2,824,739 (CI) | 21,359 | 19 | 41.9 | Antitoxin-toxin system; retron-type RNA-directed DNA polymerase; reparation DNA; HipA protein; unknowns |
|  | 36 | 2,832,452-2,838,841 (CI) | 6,390 | 5 | 39.5 | Lysine decarboxylase; transcriptional regulators |
|  | 37 | 2,862,424-2,876,659 (CI) | 14,236 | 15 | 40.3 | Acetyltransferase; oxidoreductase; unknowns |
|  | 38 | 2,878,737-2,888,713 (CI) | 9,976 | 13 | 38.7 | Antitoxin-toxin system; galactosidase acetyltransferase; unknowns |
|  | 39 | 2,894,422-2,906,822 (CI) | 12,401 | 16 | 39.5 | Glyoxalase; unknowns |
|  | 40 | 2,916,946-2,930,337 (CI) | 13,392 | 20 | 39.7 | Phenazine biosynthesis; unknowns |
| HI610 | 41 | 82,287-87,287 (CI) | 5,001 | 6 | 41.2 | Ribonuclease HI; unknowns |
|  | 42 | 410,835-423,161 (CI) | 12,327 | 13 | 38.4 | Flipasse; exopolysaccaharide biosynthesis |
|  | 43 | 625,974-635,184 (CI) | 9,210 | 7 | 33.7 | Unknowns |
|  | 44 | 2,336,720-2,344,351 (CI) | 7,632 | 7 | 30.5 | Exopolysaccaharide biosynthesis |
|  | 45 | 3,040,277-3,045,951 (CI) | 5,675 | 11 | 42.6 | Unknowns |
|  | 46 | 3,061,673-3,079,514 (CI) | 17,842 | 21 | 40.0 | Transcriptional regulator; GTPase; unknowns |
|  | 47 | 3,117,143-3,145,203 (CI) | 28,061 | 28 | 40.3 | Chaperone Tir; transporter; ATPase; phage-related elements; unknowns |
|  | 48 | 3,158,547-3,179,190 (CI) | 20,644 | 13 | 45.5 | ABC transporter; HlyD family secretion protein; RTX toxin; Thiol peroxidase; HipA protein |
|  | 49 | 3,214,269-3,225,611 (CI) | 11,343 | 14 | 40.2 | Unknowns |
|  | 50 | 3,239,567-3,266,743 (CI) | 27,177 | 23 | 39.9 | T1SS secreted agglutinin RTX; DNA-cytosine methyltransferase; unknowns |
|  | 51 | 3,301,945-3,329,885 (CI) | 27941 | 28 | 41.2 | Ribonucleotide reductase of class III; serine/threonine kinase; unknowns |
|  | 52 | 3,333,186-3,342,738 (CI) | 9553 | 12 | 41.3 | Antitoxin-toxin system; restriction system; unknowns |
|  | 53 | 3,352,431-3,362,839 (CI) | 10409 | 13 | 38.9 | Unknowns |
|  | 54 | 857,346-866,885 (CII) | 9547 | 8 | 47.1 | Multridrug transporter; RNA-directed DNA polymerase; transcriptional regulator |
| DSM21597 | 55 | 1,386,309-1,396,252 (CI) | 9,944 | 7 | 40.1 | Transporter; unknowns |
|  | 56 | 2,467,912-2,474,957 (CI) | 7,046 | 5 | 36.6 | Unknowns |
|  | 57 | 3,009,129-3,015,557 (CI) | 6,429 | 9 | 41.8 | Acyltransferase; unknowns |
|  | 58 | 3,067,727-3,076,171 (CI) | 8,445 | 7 | 36.2 | Thymidylate synthase; transcriptional regulator; unknowns |
|  | 59 | 3,129,884-3,139,439 (CI) | 10,446 | 14 | 37.7 | Unknowns |
|  | 60 | 3,149,511-3,175,115 (CI) | 25,605 | 23 | 40.9 | Abi-like protein; secreted protein Hcp; unknowns |
|  | 61 | 3,179,988-3,258,911 (CI) | 78,923 | 55 | 40.4 | GCN5-related N-acetyltransferases; zona occluden toxins; accessory cholera enterotoxin; Multiple antibiotic resistance protein marC; unknowns |
|  | 62 | 465,774-477,814 (CII) | 19,971 | 16 | 40.2 | Chemotaxis; isochomismatase; transcriptional regulator; metallo-hydrolase; transport; unknowns |
| 4299 | 63 | 1,174,041-1,188,519 (CI) | 14,479 | 8 | 41.6 | Type I restriction-modification; histidinol-phosphatase; unknowns |
|  | 64 | 1,373,012-1,383,627 (CI) | 10,616 | 8 | 40.1 | DNA-metyl transferase; chemotaxis; unknowns |
