# Supplementary material for: Comparative Genome Analyses of Vibrio anguillarum Strains Reveal a Link with Pathogenicity Traits
Source: mSystems. 2017 Feb 28;2(1):e00001-17. doi: 10.1128/mSystems.00001-17 (PMC5347184; doi:10.1128/mSystems.00001-17)
Supplement: TABLE S5 [file sys001172089st8.docx]

**Table 5S**. Distribution of virulence factors not-associated to prophages-related sequences or genomic islands in *V. anguillarum* strains

| **Strain** | **Virulence factor** | **Position (Chromosome)** | **BLASTP most significant match (accession; E- value)** | **% amino acid identity** | **Conserved domain database hit E<0.001 (accession)** |
| --- | --- | --- | --- | --- | --- |
| 90-11-286 | Toxin Fic | 1,001,267-1,002,307 (CII) | Toxin Fic *Psychromonas arctica* (WP_028869361.1; 0) | 86 | Virulence protein RhuM family (pfam13310) |
|  | Hemagglutinin | 307,796-309,796 (CII) | Hemagglutinin *Vibrio ordalii* (WP_029627190.1; 0) | 99 | Zinc metalloprotease (elastase) (COG3227) |
|  | Cytotoxic necrotizing factor 2 protein Cnf2 | 312,873-315,425 (CII) | Cytotoxic necrotizing factor 2 protein Cnf2 *Vibrio cholerae* (WP_001934618.1; 0) | 74 | None |
|  | Hemagglutinin | 2,664,802-2,668,974 (CI) | Hemagluttinin repeat family protein *Vibrio cholerae* (WP_002044679.1; 0) | 92 | Possible hemagglutinin (DUF637) (pfam04830) |
|  | Hemagglutinin | 2,668,986-2,671,097 (CI) | Hemagglutination activity domain protein *Vibrio cholerae* (WP_002044680.1; 0) | 79 | Haemagglutination activity domain (pfam05860) |
| 91-7-154 | Zonular occludens toxin | 1,795,374-1,796,753 (CI) | Toxin *Vibrio cholerae* (WP_000902276.1;0) | 76 | Zonular occludens toxin (Zot) (pfam05707) |
