# Supplementary material for: Comparative Genome Analyses of Vibrio anguillarum Strains Reveal a Link with Pathogenicity Traits
Source: mSystems. 2017 Feb 28;2(1):e00001-17. doi: 10.1128/mSystems.00001-17 (PMC5347184; doi:10.1128/mSystems.00001-17)
Supplement: TABLE S6 [file sys001172089st9.docx]

**Table 6S**. Unique prophage-related sequences distributed in *V. anguillarum* strains

| **Prophage** | **Strain** | **Size (kb)** | **#ORFs** | **%CG** | **Position (chromosome)** | **Status** |
| --- | --- | --- | --- | --- | --- | --- |
| 1 | DSM21597 | 11.6 | 12 | 45.4 | 2,718,600-2,730,249 (CI) | Incomplete |
| 2 |  | 11.2 | 11 | 44.4 | 999,802-1,011,048 (CII) | Incomplete |
| 3 |  | 8.9 | 9 | 44.4 | 1,825,738-1,834,702 (CI) | Incomplete |
| 4 |  | 8.7 | 9 | 45.0 | 863,590-877,386 (CII) | Incomplete |
| 5 |  | 7.4 | 7 | 43.0 | 3,156,125-3,163,611 (CI) | Incomplete |
| 6 |  | 7.2 | 7 | 37.9 | 1,017,841-1,025,139 (CI) | Incomplete |
| 7 |  | 6.5 | 6 | 42.7 | 1,011,149-1,017,740 (CII) | Incomplete |
| 8 |  | 5.5 | 5 | 45.0 | 3,040,100-3,045,661 (CI) | Incomplete |
| 9 | HI610 | 6.7 | 7 | 42.2 | 1,001,004-1,007,765 (CII) | Incomplete |
| 10 | 4299 | 9.7 | 10 | 42.5 | 965,738-975474 (CII) | Questionable |
| 11 | VIB93 | 20.7 | 20 | 41.4 | 249,272-270,069 (CII) | Incomplete |
| 12 |  | 16.5 | 27 | 41.9 | 1,000,399-1,016, 894 (CI) | Incomplete |
| 13 | 90-11-287 | 16.1 | 19 | 45.9 | 1,037,716-1,053,860 (CII) | Incomplete |
| 14 | S2 2/9 | 44.3 | 41 | 43.3 | 923,098-967,592 (CII) | Intact |
| 15 |  | 22.8 | 10 | 45.6 | 999,686-1,022,526 (CI) | Incomplete |
| 16 | 90-11-286 | 41.2 | 42 | 44.0 | 1,114,483-1,155,909 (CII) | Intact |
| 17 | T265 | 9.6 | 11 | 34.0 | 439,936-449,608 (CI) | Incomplete |
| 18 |  | 8.4 | 9 | 45.7 | 2,768,599-2,777,064 (CI) | Incomplete |
| 19 |  | 8.4 | 9 | 45.0 | 986,198-994,651 (CII) | Incomplete |
| 20 |  | 5.3 | 6 | 40.4 | 999,923-1,005,282 (CII) | Incomplete |
| 21 | VIB18 | 28.9 | 33 | 43.3 | 1,002,985-1,031,936 (CII) | Incomplete |
| 22 |  | 27.6 | 40 | 43.6 | 1,042,197-1,069,883 (CII) | Incomplete |
| 23 |  | 10.1 | 10 | 44.4 | 1,032,037-1,042,096 (CII) | Incomplete |
| 24 | 6018/1 | 28.7 | 30 | 40.7 | 1,098,508-1,127,222 (CII) | Incomplete |
| 25 |  | 22.6 | 23 | 44.4 | 1,057,184-1,079,828 (CII) | Incomplete |
| 26 |  | 18.9 | 17 | 44.8 | 258,780-277,708 (CII) | Incomplete |
| 27 |  | 18.4 | 22 | 42.7 | 1,079,929-1,098407 (CII) | Incomplete |
| 28 | 91-8-178 | 26.6 | 31 | 41.7 | 526,676-553,295 (CII) | Incomplete |
| 29 | 87-9-116 | 49.2 | 52 | 41.9 | 1,057,853-1,107,066 (CII) | Incomplete |
| 30 |  | 9.5 | 10 | 47.0 | 136,971-146,382 (CII) | Incomplete |
| 31 | 91-7-154 | 20.4 | 26 | 47.6 | 1,101,646-1,122,115 (CII) | Incomplete |
| 32 |  | 20.0 | 25 | 46.4 | 281,497-301,545 (CII) | Incomplete |
| 33 | 601/90 | 31.6 | 37 | 45.1 | 1,094,669-1,126,274 (CII) | Incomplete |
| 34 | 9014/8 | 27.9 | 27 | 42.2 | 701,688-729,654 (CII) | Incomplete |
| 35 |  | 28.2 | 40 | 41.7 | 1,098,754-1,127,000 (CII) | Incomplete |
| 36 | PF4 | 9.8 | 15 | 40.2 | 811,759-821,636 (CII) | Intact |
| 37 | 775 | 30.5 | 29 | 44.6 | 1,505,047-1,535,611 (CI) | Incomplete |
| 38 |  | 19.0 | 20 | 41.1 | 322,338-341,434 (CI) | Incomplete |
| 39 |  | 7.2 | 9 | 43.1 | 2,781,713-2,788,961 (CI) | Incomplete |
| 40 | PF7 | 19.0 | 21 | 42.7 | 427,555-446,557 (CII) | Incomplete |
