# Supplementary material for: Comparative Genome Analyses of Vibrio anguillarum Strains Reveal a Link with Pathogenicity Traits
Source: mSystems. 2017 Feb 28;2(1):e00001-17. doi: 10.1128/mSystems.00001-17 (PMC5347184; doi:10.1128/mSystems.00001-17)
Supplement: TABLE S7 [file sys001172089st10.docx]

**Table 7S**. Shared prophage-related sequences in *V. anguillarum* strains

| **Prophage** | **Strain** | **Size (kb)** | **#ORFs** | **%CG** | **Position^a^ (chromosome)** | **Status** |
| --- | --- | --- | --- | --- | --- | --- |
| 41 | NB10; 87-9-116; 87-9-117; 90-11-287; 91-7-154; 178/90; 601/90; 9014/8; VA1; 6018/1; VIB18; 261/91; A023; LMG12010; 51/82/2; VIB93; 91-8-178 | 53.1 | 81 | 43.1-43.7 | 1,885,577-1,935,924 (CI) | Intact |
| 42 | PF430-3;PF4 | 45.7 | 63 | 44.8 | 470,005-521,900 (CII) | Intact |
| 43 | DSM21597; T265 | 8.1 | 7 | 43.9 | 400,193-408,196 (CII) | Incomplete |
| 44 | Ba35; T265 | 9.2 | 10 | 42.9 | 986,005-995,214 (CII) | Incomplete |
| 45 | T265; 775 | 9.1 | 10 | 39.6 | 426,469-435,588 (CI) | Incomplete |
| 46 | NB10; 178/90; 91-8-178; 51/82/2 | 12.5 | 21 | 41.9 | 1,039,370-1,052,575 (CI) | Incomplete |
| 47 | VIB93; 51/82/2; 178/90; 9014/8; 6018/1; 91-7-154; 90-11-287; 601/90 | 17.9-22.4 | 21 | 49.8 | 982,382-1,000,298 (CII) | Incomplete |
| 48 | 601/90; 9014/8 | 19.2 | 19 | 41.4 | 1,036,778-1,056,390 (CII) | Incomplete |
| 49 | 51/82/2; 91-7-154 | 24.4 | 26 | 41.8 | 137,818-162,298 (CII) | Incomplete |
| 50 | LMG12010; NB10; 90-11-287; 91-7-154; VIB93; 87-9-116; 87-9-117; 91-8-178; 178/90; VIB18; VA1; 601/90 | 10.6-11.2 | 20 | 41.4 | 918,994-992,821 (CII) | Incomplete |
| 51 | DSM21597; T265 | 10.4 | 7 | 44.6 | 258,345-268,745 (CII) | Incomplete |
| 52 | DSM21597; T265 | 8.9 | 11 | 46.1 | 731,478-740,425 (CII) | Incomplete |
| 53 | DSM21597; T265; PF7 | 8.4 | 10 | 45.7 | 98,236-106,657 (CII) | Incomplete |
| 54 | 9014/8; 601/90 | 19.2 | 20 | 41.4 | 375,425-394625 (CII) | Incomplete |
| 55 | 51/82/2; T265; 90-11-287; VA1 | 17.1-19.2 | 23 | 43.4 | 1,093,915-1,110,922 (CII) | Incomplete |

^a^position according to first strain described
