# Supplementary material for: Comparative Genome Analyses of Vibrio anguillarum Strains Reveal a Link with Pathogenicity Traits
Source: mSystems. 2017 Feb 28;2(1):e00001-17. doi: 10.1128/mSystems.00001-17 (PMC5347184; doi:10.1128/mSystems.00001-17)
Supplement: FIG S3 [file sys001172089sf3.docx]

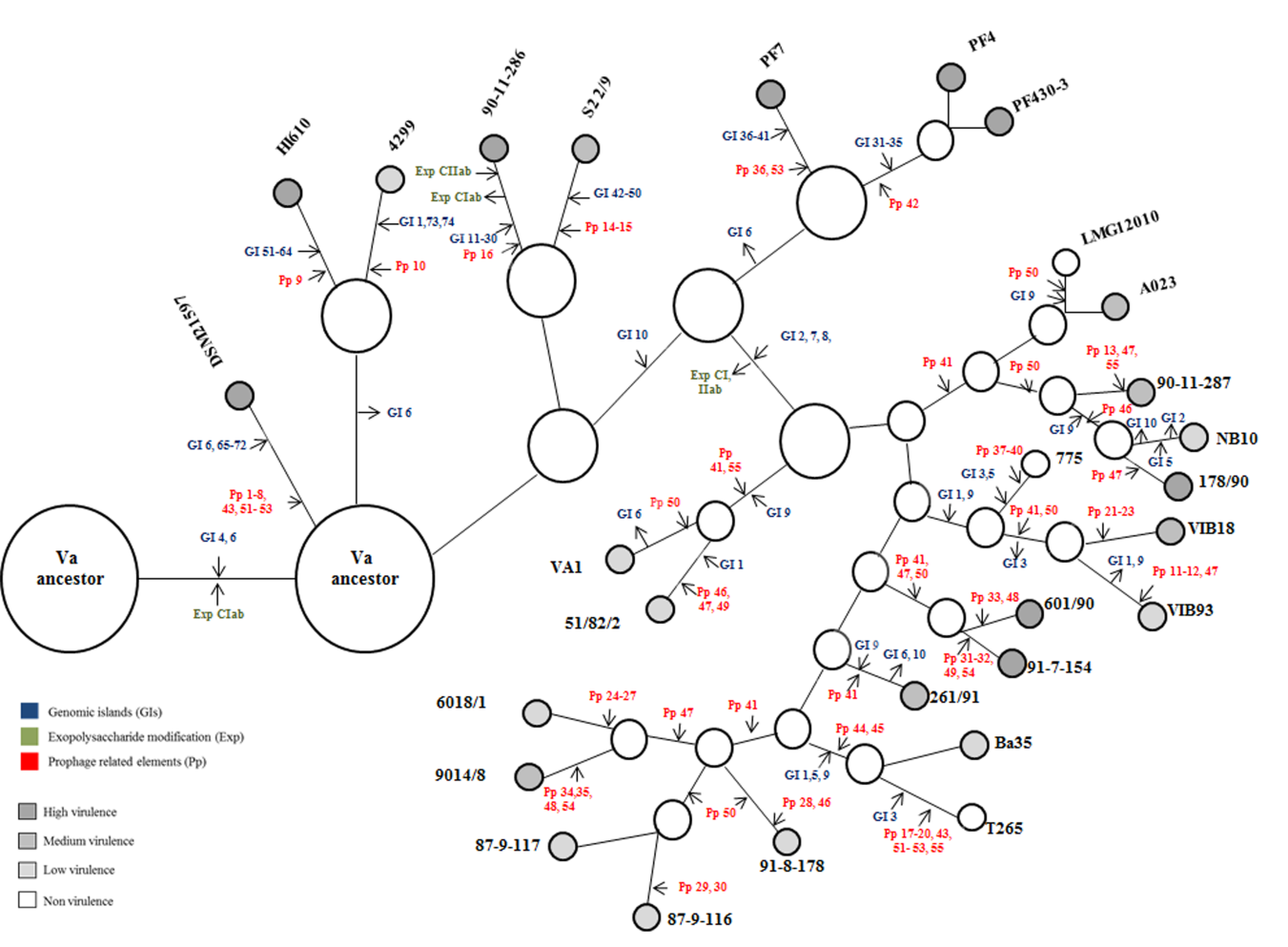


**Fig. 3S. Hypothetical evolution pathway in *V. anguillarum***. The model of *V. anguillarum* evolution suggests insertions and deletion of genomic islands (Table 4S) and the infection by bacteriophages (Tables 6S and 7S). Graphic representation of the genetic diversity is based on core genome phylogeny. Putative ancestral strains are indicated as open circles. The virulence ranking of the strains is based on three fish larval models (1).

**Reference supplementary information**

1. RønnesethA, Castillo D, D’Alvise P, Tønnesen Ø, Haugland G, Grotkjær T, Sørensen KE, Nørremark L, Bergh Ø, Wergeland HI, Gram L. Comparative assessment of *Vibrio* virulence in marine fish larvae. J Fish Dis, in press.
